# Stakeholder’s experiences of living and caring in technology-rich supported living environments for tenants living with dementia

**DOI:** 10.1186/s12877-023-03751-2

**Published:** 2023-02-01

**Authors:** Jean Daly-Lynn, Assumpta Ryan, Brendan McCormack, Suzanne Martin

**Affiliations:** 1 School of Health Sciences, Ulster University, Newtownabbey, Northern Ireland; 2 School of Nursing, Ulster University, Magee, Northern Ireland; 3 Head of Division of Nursing, Queen Margaret University, Scotland, UK

**Keywords:** Dementia, Assistive technology, Supported living, Monitoring technology, Caregivers

## Abstract

**Background:**

Technology innovation provides an opportunity to support the rising number of people living with dementia globally. The present study examines experiences of people who have dementia and live in technology enriched supported care models. Additionally, it explores caregiver’s attitudes towards technology use with the housing scheme.

**Methods:**

A qualitative research design was adopted, and eight housing schemes consented to take part in the study. A technology audit was undertaken in addition to participant interviews and caregiver survey. Seven peer researchers conducted semi-structured interviews with 22 people living with dementia. Interviews were analysed using thematic analysis. Informal and formal caregivers were invited to complete a survey to capture their attitudes towards technology use. A total of 20 informal and 31 formal caregiver surveys were returned. All surveys were input into Survey Monkey and downloaded into excel for analysis. Closed questions were analysed using descriptive statistics and open-ended questions were organised into themes and described descriptively.

**Results:**

The technology audit identified that technologies were in place from as early as 2002. Technology heterogeneity of, both passive and active devices, was found within the housing schemes. Technologies such as wearable devices were reportedly used according to need, and mobile phone use was widely adopted. The themes that developed out of the tenant interviews were: *Attitudes and Engagement with Technology; Technology Enhancing Tenants Sense of Security; Seeking Support and Digital Literacy; and Technology Enabled Connection*. A lack of awareness about living alongside technology was a major finding. Technologies enabled a sense of reassurance and facilitated connections with the wider community. The interaction with technology presented challenges, for example, remembering passwords, access to Wi-Fi and the identification of its use in an emergency. The caregiver survey reported a range of facilitators and barriers for the use of technology within care. Both types of caregivers held relatively similar views around the benefits of technology, however their views on issues such as privacy and consent varied. Safety was considered more important than right to privacy by family caregivers.

**Conclusions:**

The present study provides new insight into stakeholder’s experiences of living, working and caregiving alongside technology in supported living environments. As the generation of people living with dementia become more tech savvy, harnessing everyday technologies to support care could enable holistic care and support the transition through the care continuum. Advance care planning and technology assessments are at the very core of future technology provision. It is evident that a paternalistic attitudes towards technology use could impact the multitude of benefits technology can play in both health and leisure for people living with dementia and their caregivers.

**Supplementary Information:**

The online version contains supplementary material available at 10.1186/s12877-023-03751-2.

## Introduction

The global population of people living with dementia is estimated at 46.8 million and expected to double every twenty years [1]. Greater demands for holistic person centred care is required now and for the future [2]. Dementia is a term for a cluster of symptoms that degenerates the cells in the brain leading to impaired memory, thinking, problem solving and language. It is a complex condition because each presentation is unique to the person and their needs change overtime. Internationally, innovations in person centred care provision, drivers for earlier diagnosis, environmental design and housing have created opportunities to support people live with autonomy on their dementia journey. Equally, the voice of people living with dementia has strengthened amplifying their needs and gaps in research [3]. Technology has been proclaimed as a solution to support current care provision and an abundance of innovation has emerged over the last twenty years [4, 5]. The complexity of this research field means it can often be difficult to identify the useful solutions for people living with dementia. The purpose of this research is to explore the experiences of people living with dementia, and their caregivers, as they live in technology-enriched supported living environments.

Within the United Kingdom, government, industry and research investment has been considerable to drive innovation of technology to improve well-being, independence and length of life [6]. Policies such as the ‘*Challenge on Dementia 2020*’ focused on improving care, increasing research, and raising public awareness in England. Equally, European research funding schemes have sought to capitalise on technology advancement for public health. The World Health Organisation Global Action Plan on the Public Response to Dementia (2017–2025) specifically sets out that technology is a core principle in the provision of dementia care and advanced planning. Guidance from the National Institute for Health and Care Excellence (NICE) sets out person centred care at the heart of dementia service provision [7]. Technology within dementia care needs to balance technology as a care approach and the personhood of those living with dementia [8]. Person centred practice has parallels with recent research in human computer interaction and technology design for and with people living with dementia [9]. Adopting a critical dementia lens to technology provision focuses on the unique experience of the dementia journey from a social constructionist point of view [10]. This viewpoint considers technology as a tool for empowerment and recognises the unique needs, sense of self and potential of people living with dementia [11].

Assistive technology or technology can be defined as a system or device that enables the person to complete a task, they would not be able to do at all or with ease without it. It is a very broad term, particularly when it is applied to the various ways and devices that could be used to support people living with dementia. It describes a vast spectrum of systems from stand-alone devices to complex ubiquitous smart home environments [12]. Assistive technologies can be classified as being used ‘by’ the person living with dementia, ‘with’ the person living with dementia, or ‘on’ the person living with dementia [13]. User activated technology refers to devices the person engages with such as a touch screen device, call systems to seek help, mobile phones, and therapeutic interventions. Passive or pervasive technology are monitoring systems used ‘on’ people such as sensors to detects motion, pressure, inactivity, falls and temperature. This technology does not require any active input from the user and sends signals to the caregiver when assistance is required. Localisation technologies are generally wearable, like a pendant or bracelet, and support locating a person if they get lost [14]. Automatic prompts and reminders like medication dispensers and electronic calendars can be useful solution to use with people living with dementia. Everyday technologies such as virtual assistance like Amazon’s Alexa can support orientation (*Alexa, what time is it?)* and the activation of devices such as lights and television. Equally, the marketplace has a range of smart watches and smart phones. Assistive technology is more commonly used for safety as opposed to a device for well-being and leisure [15].

In 2016, Gibson and team reported 1.7 million telecare users and 171 different technology products [13]. The wide spanning variation of devices and the limited voice of people living with dementia was a common theme reported in systematic literature reviews in this field [16–19]. Additionally, a number of useful prototypes reported in research have not made it to the market place [20]. The biggest barriers for assistive technology adoption are people living with dementia not wanting to use it [21], cost [21] access [22] and digital literacy [23]. Recent reviews indicated the need for robust research to support the adoption and use of solutions for everyday living [4, 5]. Furthermore, research is required to determine the benefits of customisable solutions to support people throughout their dementia journey [17]. This has implications for clinical practical and care provision as it is difficult to determine the usefulness and user-friendliness of assistive devices and telecare for people living with dementia and their caregivers.

There are no guidelines on the provision of assistive technology in dementia care. ATTILA, a randomised control trial, found that assistive technology provision for people living with dementia ageing in place is not in line with best practice as the provision of technology often does not follow the recommendations made by assessors [15]. Further research is required as no psychological impact was found within the informal caregiver group when assistive technology and telecare was used to compliment care at home [24]. The findings also indicated that assistive technology and telecare did not necessarily maintain ageing in place for longer [2], highlighting the importance of innovation in housing models.

Living at home for as long as possible is enshrined in policy and supported by technology provision [1]. Where a person lives is intrinsically linked to their personhood and essential to support quality of life [25]. Therefore, it is important to consider the environment, along the continuum of care, that is appropriate for a person to move into when community living is no longer a feasible option. Supported living provides an alternative housing model and incorporates dementia design principles and person centred practice into its ethos [26]. Different terms are used to describe this housing model such as extra care housing [27], assisted living [28] and small home like facilities [29]. Telecare and assistive devises are often used to support independence [30] and foster person centred care [31]. The housing model offers domiciliary care according to need and informal caregivers are expected to maintain a caregiving role in collaboration with formal caregivers. Supported living aims to offer independence and autonomy as a community based housing model, however, a recent literature found this is not always achieved due to lack of resources [32]. Limited research has considered the potential of technology to support tenants independence and autonomy within this housing model [33].

Technology intervention can be an asset to the caregiving role [2]. Both informal and formal caregivers can benefit from the innovation [34], especially as caregiving changes during the transition from community to supported care environments [35] and throughout the dementia care pathway [36]. Equally, research illustrates that caregivers play a key role in enabling active use of technology in the lives of people living with dementia [37]. Informal caregivers in the community are more likely to be using technologies in their everyday life and as a result have the digital literacy to incorporate assistive technologies into supporting care provision [38]. Gullslett and team [39] explore next of kin experiences of monitoring technology in residential setting and highlight the different needs, information and expectations each caregiver will have in terms of the use of such devices. The importance of collaborative practice was also highlighted as essential. The challenges identified was providing informal caregivers with information that met their needs and digital literacy levels [39]. Assistive technology can support formal caregivers reduce the burden of care and uphold their duty to protect people from harm [40]. The devices were found to support formal caregivers to react at speed when required and enable people living with dementia to live independently at their own pace [41]. Research has identified challenges such as alarm fatigue, device maintenance and the lack of robust design to withstand the daily routines within a long term care setting [42]. In addition, the multiple dimensions of the environment as a workplace, a home and a care provider present ethical issues [40]. The concept of surveillance and workplace oversight can be a barrier to formal caregiver adoption [42]. Assistive technology did not appear to impact the decision to move into supported living settings, however it was valued as a support system by informal caregivers post transition [43].

The landscape of the care environment changed rapidly as a result of Covid-19 [44]. During the pandemic, people living with dementia were at risk of social isolation during periods of restricted measures [45] and digital technologies provided potential for social connectedness, particularly with families and friends outside of the care environment [46]. Covid-19 created rapid change in the way technologies were used and exacerbated the gap between technology adopters and those with low digital literacy [11]. In terms of care provision, it was found that technology supported communication between formal and informal caregivers to support the emotional well-being of people living with dementia in long term care setting during the pandemic [47].

Even with wide spread innovation in this field, there is limited research into technology design, delivery and experiences in supported living environments [48]. The role of technology for people living with dementia and other stakeholders became even more important during a global pandemic [11, 47]. Research highlights the extensive ethical debate around the use of monitoring technologies [49, 50]. However, little is known about the first-hand experiences of those living alongside technology that has the potential to be use ‘with’, ‘by’ and ‘on’ them and their caregivers.

### Aims

- To examine the experience of living in a technology-rich environment by people who have dementia that live in these settings.
- To explore stakeholder’s attitudes towards technology use in the housing scheme

## Methods

### Research design

The purpose of the study was to capture in-depth understanding of technology enriched support housing from the perspectives of three stakeholder groups: the tenants living with dementia, their informal caregivers, and formal caregivers. A qualitative study with older people as peer researchers was adopted. Peer researchers were recruited and trained to conduct the interviews with tenants living with dementia. The focus of this paper is to report on the perspectives of technology from all stakeholder groups. Ethical approval to conduct the study was granted by the Research Office of Ethics Committees of Northern Ireland (ORECNI) under REC Reference 15/NI/0160. The research activity took place between 2015 and 2019.

### Recruitment

Twelve housing schemes that were recorded as providing supported living for people living with dementia in a technology enriched environment were identified through a government department. A technology enriched environment was where a range of telecare and assistive devices such as alarms, sensors, and monitors, were embedded into the living environment to support care. The housing schemes were registered as having technology as part of the care provision with government agencies and this was confirmed through a technology audit by the research team. An information pack including consent forms and scheme participant information sheets were posted to the eligible housing schemes. One scheme responded that provision was not for people living with dementia. Eleven housing schemes expressed interest. A site visit to each housing scheme to identify if they met the inclusion criteria (Table 1) excluded one site due to the nature of care provided as it was not defined as supported living and a second site decide not to commit due to time. Site H was part of the project up until the tenant’s interviews when it became clear that people living with dementia were no longer living in the supported living section of the scheme.

**Table 1:** Inclusion and exclusion criteria

Housing Scheme	Tenants	Informal Caregivers	Formal Caregivers
Supported living for people living with dementia Technology enriched environment to support care Manager willing to give consent	Living in the housing scheme more than 6 months A diagnosis of dementia Willing to give consent according to process consent approach	Over age 18 Caregiver to tenant Willing to give consent	Over age 18 Working at the facility Willing to give consent

The recruitment of tenants, informal caregivers and formal caregivers were from the schemes that consented to their involvement in the project. Tenants were recruited by the scheme manager in partnership with the research team through purposeful sampling. Process consent (Dewing, 2008) was adopted and aligned to the approach used daily with the tenants. Scheme managers consulted with family members and tenants provided consent for themselves. Written consent was viewed as valid at the time the trusted staff member went through the participant information sheet to explain the study and obtain written consent. On the day of the interview, verbal consent was sought, and visual cues were observed to ensure an on-going, fluid approach to consent was adopted. Recruitment for the survey was undertaken through the housing schemes after the one-to-one interviews were completed. Informal caregivers and formal caregivers could complete an online version held on Survey Monkey or a hardcopy survey. The links to the online survey were sent to the scheme manager to be distributed to both staff and family and friend caregivers. The hardcopy of the survey was left at the entrance to the housing scheme for a six-week period with a box to submit the survey after completion.

### Sample

A total of eight schemes completed all aspects of the project. Twenty females and two males, with a confirmed diagnosis of dementia participated in the interviews. One female was under sixty (Susan) and lived with her husband. Participants were not asked specific demographic information in line with recommendations set out for inclusive engagement with people living with dementia in qualitative research [51, 52]. Two interviews were excluded after it became clear the individuals were not living in an environment with technology to support their care. Informal caregivers completed *N* = 20 surveys and formal caregivers completed *N* = 31.

### Data collection

#### Technology audit

The Technology Audit Tool was developed as an outcome from the systematic review undertaken in the project [33]. Due to the breadth of data to capture from the schemes and the complexity of terms for non-expert users in this field, it was decided to divide the audit into two parts. The first part was a template (included in supplementary information) for completion by a designated person (scheme manager or senior member of staff) to complete in their own time and email it back to the research team. A follow-up phone call was then made to the designated person to discuss the outcome of audit form A and complete part B (included in supplementary information) over the phone. A total of eight schemes completed audit form A and four completed Audit form B. All data was included even if the second part of the audit was not completed.

### Tenant’s perceptions of technology

Semi-structured interviews were undertaken with tenants facilitated by peer researchers. Peer researchers were seven older people, with experience of caring for people with dementia, that used their experiential knowledge to facilitate the interviews. Details of the peer researcher role and experience was set out in a separate paper [53]. A total of twenty females and two males, with a confirmed diagnosis of dementia participated in the interviews. The interviews were held at a time and place decided by the tenant, in collaboration with a staff member within the housing scheme. Each interview began in the same manner. The first author of this paper and peer researcher were introduced to the participant by the member of staff in the location for the interview. The staff member outlined the purpose of the interview and the tenant confirmed they were still happy to take part in a one-to-one interview and consent to the use of a voice recorder. The researcher then introduced the project, and her and the role of the peer researcher. Once the voice recorder was switched on, the peer researcher took the lead in the interview and asked questions based on the topic guide. Questions were focused on the general experience of living in a supported living environment and the technology within the environment (Table 2). The interview was guided by CORTE guidelines [52]. The peer researcher aimed to build a rapport with the tenant and reflected on their own lived experience at times. The first author kept a diary of the interviews to capture her own experiences and feelings and those of the peer researcher following a debrief.

**Table 2:** Technology focused topic guide questions

If you need help, how do you get it?
Have you any technology in your home like alarms or intercom speakers?
*(If you see any technology in the environment point to it and ask if they use it.)*
Does anything help you remember your appointments?
How do you contact your family?
Have you ever used a computer, laptop or tablet?
Have you ever worn a bracelet or necklace that has an alarm in it you can press in an emergency? Have they ever pressed it?
If there is an Intercom: How do you feel about it going off?
Have you ever heard any alarms going off? Do you know what they are for?
*These questions were asked as part of a larger interview and therefore not asked in the above sequence*

### Informal and formal caregiver survey

A cross sectional survey of formal and informal caregivers exploring the use of electronic assistive technology in supported living environments was developed for this project. The items were generated through a systematic literature review [33] and interviews with informal caregivers (IC) (*N* = 25) [43] and formal caregivers (FC) (*N* = 21) [41]. The survey included nine Likert type data items and open-ended questions (ten within IC version/ eleven within FC version) delivered as a self-administered survey through an online platform (Survey Monkey) and hard copy distributed within the housing scheme. The development of the survey was first described in the final report [54]. The Likert type data items ranged from strongly disagree, disagree, neutral, agree, strongly agree. No attempts were made to combine the responses into a scale to create a score. Statements were balanced accordingly, so *N* = 3 items were positive, *N* = 4 items were negative and *N* = 2 were neither positive or negative. The Likert statements were the same in both IC and FC surveys to enable a comparison of their perceptions towards the use of technology and are presented in Table 5 in the results section. Demographic questions and open-ended questions were set out at the beginning of the surveys. Open ended questions included: Are there any advantages to the use of technology to support your friend or relative (Yes/No)? Please explain your answer. Do you think tenants should have access to the internet? Does technology impact your caregiving role? Finally, an explanation of the term technology was outlined at the beginning of the questionnaire to ensure each participant was aware of the meaning within the current project.

### Data analysis

The data from the Technology Audit was extracted from the forms, input into excel, categorised, and presented descriptively. All survey responses were input into Survey Monkey and collated onto excel. The responses from IC and FC were populated separately. Closed questions were analysed using descriptive statistics. The open-ended questions were organised into themes and described descriptively. Finally, the data from the Likert statements were compiled according to the type of caregiver and presented in Table format.

All recorded tenant’s interviews were transcribed verbatim and input into a qualitative data analysis computer software package (NVivo 11). Each interviewee was given a pseudonym. Thematic analysis was used and started with the process of familiarisation, as set out by Braun and Clarke [55, 56]. The six phases for analysis: familiarising yourself with dataset, coding, generating initial themes, developing initial themes, developing and reviewing themes, refining, defining and naming themes and writing up, were followed. Deductive coding, using both semantic and latent codes, were generated by the first author during two rounds of coding. The codes and the coded data were examined independently to generate patterns of meaning and themes. The candidate themes were then discussed within the wider team (all authors). A workshop was conducted with peer researchers to collaboratively interpret the data. The peer researchers were asked to read the transcript of the interviews they completed and highlight the important components. A group discussion generated and consolidated the themes further in advance of the group exploring the themes the research team had developed. The similarities and differences were highlighted, and themes were further developed. The themes were checked against the entire dataset and named. This approach enabled a process of peer validation and opportunities to reflect on the findings in the team, including the peer researchers. Rigor was maintained throughout the analysis through journaling, checking interpretations with peer researchers and maintaining one researcher through all data collection and analysis. Rich quotations are used with the intention of maintain the accuracy of the voice of participants.

## Results

### Technology Audit

A total of eight schemes completed audit form A and four completed Audit form B. The earliest technology enriched scheme was opened in 2002, while the most recent was opened in 2014. Four of the schemes emphasised the advantages of the technology in terms of the independence and security it provided (for example ‘*Technology is used within Site C to ensure the safety of tenants which minimises staff intrusion and maximises privacy and independence’* Site C).

The housing schemes highlighted the ethical use of technology with human rights, privacy, and dignity prioritised for tenants.

The use of the technology was personalised according to choice and the specific needs of tenants. This non-restrictive approach to technology use aimed to support the independence, safety, well-being, and health of tenants (for example ‘*The technology allows tenants to remain independent with support, safety and security that does not restrict their movement in and out of the scheme’* Site A).

The audit illustrated a heterogeneity of technologies within the housing schemes. Three out of the four schemes that responded to Audit B stated that their technology systems were bespoke for their scheme. The themes of safety and risk management resonated through the audits, as did the need for personalised and person-centred approaches (for example ‘*You can go too far with technology –it all should be looked at in its own merit. The non –intrusive is lovely –and we need it with the person centred –you need to be present –to be with the person’* Site B).

One facility pointed out the need to modernise the technology as it remained unchanged since its inception more than ten years ago (for example ‘*The technology in the scheme is good with consideration of the age (13 years). However, certain systems that would have been great, now have since failed and not been replaced (intercom system from front gate into each individual bungalow). The technology in the scheme that is useful is the intercom system to our handsets, however, this is also starting to fail and will need replaced*’ Site E).

Table 3 sets out the types of technology used within each of the housing schemes. For example, Close Circuit Television (CCTV) was used in five settings and intercom systems in the tenants living space were used in seven settings. This enabled tenants to speak with staff through a handset as well as staff received alerts from movement sensors and call requests. In two housing schemes the intercom system supported the tenants to view the person ringing their door bell. Site I felt ‘*there is (an intercom) but it’s not used very often because it is confusing for tenants*’. Technology for example sensors on beds and chairs, and fall detectors were reportedly used when a tenant needed to use these devices as opposed to every tenant in the housing scheme. Enuresis/continence sensors were not reported.

**Table 3:** An o﻿verview of TESA and participants perceptions of technology

Housing Scheme	Technology Description by Scheme in Audit	Tenants
**Site A** **Year opened:** 2012 **Capacity:** 35 **Occupancy at time:** 30	•CCTV is used in the communal areas and entrance to scheme •Sensors are used on the door of tenant’s flats and side door of scheme •Tenants use a pull cord to seek assistance •Tenants receive an immediate response through an intercom •Alarm bracelets are available according to need •A range of technologies such as mobile phones, iPads and memory aids were used by specific tenants	**Bridget:** Has mobile phone. Aware of pull cord, intercom system and of pendent but doesn't use it
		**Susan:** Aware of technology available. Uses iPad and smart phone. Has made plans for future use of bed and door sensors if her night wander walking progresses
**Site B** **Year opened:** 2005 **Capacity:** 30 **Occupancy at time:** 29	•Intercom and doorbell at entrance •Tenants use a pull cord to seek assistance •Phone is used in bedrooms not intercom •Motion sensors and door sensors are used in bedroom with consent •Bed sensors and electronic tracking (GPS) used according to need •Scheme has seven tablets for tenants to use •Data informs care plan	**Ann:** Knows to press button to call for assistance
		**Emma:** Phone by bed to call for assistance. Uses mobile phone
		**Elizabeth:** She was aware of phone and call button over bed
**Site C** **Year opened:** 2002 **Capacity:** 25 **Occupancy at time:** 25	•Keypad at entrance •Tenants use a pull cord to seek assistance •All tenants have fall detectors, motion sensors, inactivity sensors, motion sensitive lights, and pressure sensors on bed and floor •Electronic tracking and pendant or bracelet alarms used according to need •Data informs care plan	**Sally:** Uses mobile phone and pull cord
		**Elma:** Uses mobile phone, pull cord and call button
		**Michael:** Wears a pendant alarm
**Site D** **Year opened:** 2014 **Capacity:** 30 **Occupancy at time:** 25	•Doorbell and keypad at entrance •Tenants use a pull cord, wearable devices and fixed button on wall to seek assistance •All tenants have fall detectors, motion sensors, and pressure sensors on bed and chair •Pendant or bracelet alarms were used according to need •Staff respond to alerts immediately through intercom	**Helen:** Uses a mobile phone, pull cord and intercom system
		**Clare:** Uses a bracelet to call for assistance
		**Mary:** Not aware of technology
**Site E** **Year opened:** First in 2004 and new section in 2009 **Capacity:** 23 **Occupancy at time:** 23	•Keypad and key card at entrance •CCTV at entrance, side door and communal areas •Tenants use a pull cord and wearable devices to get immediate assistance •Pendant or bracelet alarms used according to need •Specified tenants have a computer, iPad and mobile phone	**Marie:** Discussed the pull cords, intercom system, and landline phone to call staff
		**Sadie:** No awareness of technology. Phones staff to get assistance
**Site F** **Year opened:** 2008 **Capacity:** 12 **Occupancy at time:** 12	•Fingerprint enabled keypad at entrance •CCTV at front and back entrance •Tenants use a pull cord and fixed button on wall to get immediate assistance •Pendant or bracelet alarms and bed sensors are used according to need •Data informs care plan	**Aoife:** No awareness of technology
		**Jennifer:** No awareness of technology
		**Sarah:** No awareness of technology
**Site G** **Year opened:** 2005 **Capacity:** 61 **Occupancy at time:** 54	•Keypad and keycard at entrance but tenants do not have free movement in and out •No CCTV •Sensors at side door and front door •Tenants use a pull cord and wearable devices to get immediate assistance •All tenants have fall detectors, inactivity sensor, and pressure sensors on bed, floor and chair •GPS and Pendant or bracelet alarms are used according to need •Data informs care plan	**Celine:** No awareness of technology
		**Denise:** Has a mobile phone and a computer and disappointed no Wi-Fi so can’t email or Skype. Uses pendant and intercom system
		**Niamh:** No awareness of any technology
**Site I** **Year opened:** 2001 **Capacity:** 35 **Occupancy at time:** 30	•Fingerprint enabled keypad at entrance •No CCTV •Door sensors for tenants at night •Tenants use a pull cord and wall fixed buttons •Report don’t use wall button or intercom due to capacity •All tenants have fall detectors, motion sensors, inactivity sensor, and pressure sensors on bed •Pendant or bracelet alarms are used according to need •Communal computer supervised use •Snoezelen room •Data informs care plan	**Louise:** Wearing a pendent but not aware of use
		**Stephen:** Uses a pull cord but stated he would not use a pendant as he doesn't need one and wouldn't use the intercom system

Wearable devices were available in most schemes however were only worn by tenants when a need was identified. The sites that reported availability of certain wearable devices are outlined in Table 4. One scheme stated, ‘*wrist pendants are a choice made by tenants and their families but encouraged by the scheme*’ (Site E).

**Table 4:** Wearable devices worn by specified tenants

	Site A	Site B	Site C	Site D	Site E	Site F	Site G	Site I
Electronic Tracking Device (GPS)	P	P	P				P	
Alarm pendent			P	P	P	P	P	P
Alarm bracelet	P		P	P	P	P		P

**Fig. 1:**
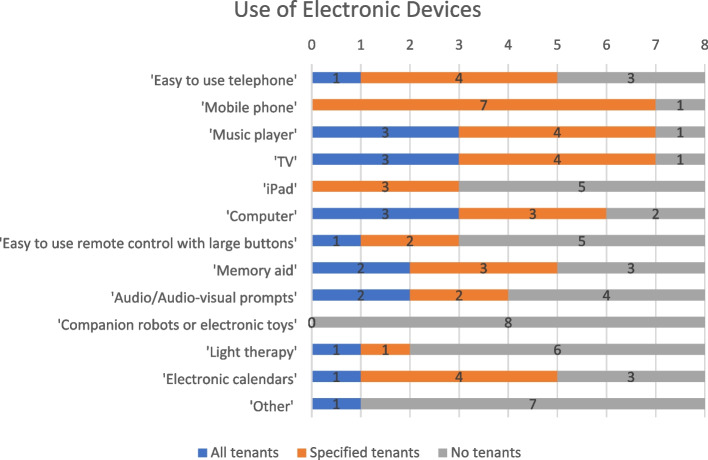
Electronic devices used by tenants

In Fig. 1 the digital devices used in schemes by tenants were highlighted. A mobile phone was commonly reported. The other device available in Site I was the Snoezelen room.

Tenants consented to the technology within the scheme during the orientation following taking up tenancy. Methods of seeking routine and emergency support from staff members were through the same approach via fixed wall buttons, pull cords and wearable devices (for example ‘*There is no difference because the technology is used as a preventative measure rather than a reactive one*’ Site I). Mobile phones and pagers were used by staff to receive notifications from tenants.

The data was used by schemes in different ways. For example, to identify tenant’s physical activity level and monitor sleep wake pattern. Two schemes reported the data useful as an indicator of changing health status by observing a ‘*shift in pattern of movement’* (Site C) and ‘*invaluable in detecting a UTI*’ (Site B). In addition, the technology was usefully for night-time monitoring (for example ‘*The technology would inform more in the evenings – peoples movement at night – and on the stairs – during the day it is not really in use. The alarms up into the bedrooms. There are some things are timed for example the water left on – this will come through to the handset*’ Site B).

### Tenant’s perceptions of technology

The themes that derived from the analysis were: *Attitudes and Engagement with Technology; Technology Enhancing Tenants Sense of Security; Seeking Support and Digital Literacy; and Technology Enabled Connection*.

### Attitudes and Engagement with Technology

Interviews revealed a mixture of attitudes towards the technology in the surrounding environment from not being aware to a vital aspect of independent living and the ability to connect with others. Participants described a range of devices during the interviews such pendant alarms, pull cords, buzzers, intercom systems, computers/ laptops, tablets, and mobile phones. The types of devices ranged from personal electronic devices for social interaction, to wearable devices to seek formal support, communication systems and low-tech alerts systems like pull cords. A key use of the devices mentioned was for seeking support from formal caregivers. The lack of awareness about the technology in the surrounding environment was a major finding within the tenant’s interviews. A total of eight participants had no knowledge of the technology at the time of the interview and were passive users of the pervasive technologies used ‘on’ them.

‘***Peer researcher***: *Do you have any sort of system in the flat that you can use to call the staff?*

***Celine***: No. No. I don’t have anything like that.

***Peer researcher***: No wee emergency buttons?

***Celine***: No’

(*There was an intercom system and pull strings visible in Celine’s environment*).

The findings were aligned to the nature of technology as pervasive and unobtrusive within the supported living environment. When participants were asked about technologies and alarm systems, they regularly spoke about testing the fire alarm in the scheme. On the other hand, two participants had significant knowledge of technology in their surrounding environment and of digital devices including the use of computers and tablets. The decision to move into the housing scheme was based around the technology provision offered. Susan felt that the technology in the supported housing would provide a support system to remain independent, safe, particularly at night-time, and an active citizen for longer.

‘*I didn’t feel safe on my own environment (own home with husband) so I was looking for somewhere to make me feel safe and secure*.’ **Susan.**

Twelve participants had varying degrees of basic knowledge around technologies, primarily used to support their care. Many participants referenced the pull cord that was part of routine care provision as opposed to a unique system characterised by the telecare and monitoring technologies embedded in the environment. These participants tended not to use technology beyond seeking caregiver support. This required some active engagement with the devices in their surrounding environment.

### Technology enhancing tenants sense of security

Participants were living in an environment with pervasive technologies used to support care. It was evident that overall, within the technology enriched housing schemes, tenants felt safe and maintained a sense of privacy. ‘*More than anything you have an awful lot of privacy. If you want your privacy you get it.*’ **Emma**. Wearable devices were used by some tenants as part of their care plan. The wearable devices were both acknowledge and unacknowledged while worn during the interview. It was evident from the people who were aware and used the technology in their environment that it gave them a sense of security knowing there was immediate access to support when required. The ability to request support from the privacy of their own space enabled independence and autonomy.

‘*I feel comfortable because I know there’s always somebody here if I need anything*.’ … ‘*I feel secure.*’ **Clare.**

Wearable devices also enabled community engagement and the ability to leave the housing scheme. This further extends the freedoms of the tenants and provides a mechanism to access a level of support beyond the housing scheme. Bed monitoring sensors alerted formal caregivers when tenants got out of bed. Participants spoke about the comfort this provided, particularly when they woke in the morning, and it also gave peace of mind if nightwalking. This enabled caregivers to be notified to check on the tenant or have an interaction through an intercom. One participant spoke about how when she gets up staff would ask her how she was through the intercom.

‘*They would speak through (intercom) sometimes in the morning. There is something here that alerts them. It’s here below the bed. …..It’s a good thing because it makes you feel safe*.’ **Denise.**

Falls were reported frequently within the interviews and were often cited as the reason the tenant had transitioned into the housing scheme. This created a level of apprehension around future falls and independent living. Technology was reported as reducing this apprehension. It could be used in an emergency to seek out formal support and this gave reassurance to those who would need additional support.

‘*That’s what I have the cord for if I needed anything or if I fell, I’m always very prone to falls. But then I don’t mind, I don’t mind falling now because if I fell or anything that’s what that there’s for, I just pull that*.’ **Clare.**

Decision making for future care needs was possible and reported in this environment. One participant spoke about her choices for using technology in the future as her dementia journey progresses. She had discussed and given her consent to the scheme for the future use of sensors on her doors as she was particularly concerned about night-time walking. When asked if she felt the use of technology would have an impact on her privacy, she responded:

‘*I don’t personally to be honest I think if it keeps you safe.’* **Susan.**

### Seeking Support and Digital Literacy

The way in which a person interacts with the technology in the surrounding environment was an important pattern in the data. This theme reflected the tenant’s awareness of technology in their lived environment and their literacy to use the systems when they need to. It was also reflected in the tenants decision making to engage or not to engage with the devices to seek support. Typically, pervasive systems do not require active input from the user. Many participants were unsure of the use of devices when pointed to in their living environment. This becomes a challenge when the tenant was required to use the device to seek support.

‘*I fell about 3 weeks ago, clumsy, footless, that’s all it was. Pressed the button on the wall and it’s not because she’s here again now, but within 2 s 2 girls up to see if I was alright*.’ **Ann.**

User activated technologies required the person living with dementia to remember to use such devices if they needed support urgently. The challenge is for a person with cognitive impairment to remember how to request support when they need it.

‘*Well at the time I fell in the bathroom, I was on the toilet and I got up and my head went dizzy and I fell. But the way I fell I never remembered about the thing you, the strings you pull, you know.’* **Bridget.**

In addition, participants reported choosing to interact with devices or not. A commonly reported reason for not using the available technology to seek support within the tenant’s flats was because they did not want to disturb the staff.

‘*I just don’t like it, it’s me, I can’t go and press it because I think they’ve enough to do without coming up to me*.’ **Helen.**

Contrasting this view, some participants report using user activated devices to demonstrate their knowledge and ability to use the surrounding technologies.

‘*I always use mine. Even if it’s only for a simple thing, I find it’s better to let the staff know that you’re using it and you know how to use it*.’ **Elma.**

### Technology enabling connection

It was evident that technical devices kept participants connected both internally in the scheme and externally with family caregivers and friends. Monitoring devices such as wearable pendants and fixed alert systems enabled tenants seek out social and functional support from formal caregivers. This enabled autonomy, privacy, and independent living alongside feeling connected to people that provide support.

‘*If you need somebody, some help you’ve only got to talk there and ask for help if you need, if you had a fall or anything you can ring, cords to pull, you know*.’ **Bridget.**

Mobile phones were the most popular personal device reported. The use of mobile phones enabled the maintenance of strong relationships with important social contacts such as families and friends.

‘*My best friend is my mobile*.’ **Emma.**

Everyday technologies such as computers, laptops and iPads were reported as a supportive mechanism to stay connected. These types of digital devices were infrequently reported however it was often when family members lived abroad. Another person used their iPad to engage with a group developed to empower people living with dementia and engage in citizenship.

‘*I got this as a present (iPad) it is so easy it makes everything so easy you know for me now.*’ **Susan.**

Susan outlined that apps such as Dragon dictate were useful to use speech typing as opposed to touch typing, particularly when writing was no longer possible. During the interviews, participants in two separate schemes mentioned iPad training encouraged by staff within the scheme.

The cognition required to actively engage with technology was reported as a challenge in relation to digital devices that enabled connection. For example, it was difficult to remember passwords to log onto computer and the various accounts for socialisation such as Skype, email, and social media.

*‘I find Skype easier than email, I can Skype them, but he (her son) has set me up with a password and I have forgotten’*. **Denise.**

There was also a belief that technology was not something tenants were accustomed to and therefore, not relevant at this point in their life. Particularly, digital technologies to enable connection were not viewed as important to interact with. ‘*I’m not a computerised person*.’ **Michael.** There was a sense of satisfaction with the care provision within the scheme and needs been met, therefore technologies that support connecting to outside the housing environment were not required. For these individuals, they felt connected within the scheme.

One barrier to technology supporting connectedness was that Wi-Fi was not widely available to the tenants. The lack of Wi-Fi, unless purchased personally, really limited access to online services that would enable communication between the tenant and their friends and family.

*‘They have Wi-Fi in lots of places but they don’t have it here. I don’t know why.*’ **Denise.**

### Cross sectional survey of formal and informal caregivers

A total of *N* = 20 family and friend caregivers from six of the eight recruited schemes completed the survey, of which 80% (*N* = 16) were female and 20% (*N* = 4) were male, proving care for 5.2 years on average. A total of *N* = 31 staff caregivers, with years caregiving experience on average, from four of the recruited schemes completed the survey, of which 19.35% (*N* = 6) were male and 25 (80.65%) were female. Training was usual reported as an induction activity, with 67.74% (*N* = 21) staff reported completing it. Alarmingly ten staff members (32.26%) reported never having training to use the technology systems in the housing schemes.

Informal caregivers widely reported the positive aspects of technology to support care (90%/*N* = 18). In addition, 88.89% (*N* = 16) did not see any negative reason around technology use. In advance of the tenants move to the schemes, six participants (31.58%) were influenced by the technology available during the decision making process. Participants cited reasons such as ‘*I liked the way they use all the available aids such as sensors beside the bed to indicate the patient is out of bed,* the technology *‘is useful for safeguarding’,* ‘*thought it was more up to date*’ and ‘*the degree of vigilance it gives’*. It was also reported that ‘*quality of care’*, ‘*the secure setting’*, ‘*the care was needed regardless of what technology was available*’, were among the reasons the scheme was selected as a housing option for their loved one. Technology was reported to impact the caring role for 80% (*N* = 24) of staff caregivers. Table 5 sets out an overview of the advantages and disadvantages outlined by caregivers.

**Table 5:** Perceived facilitators and barriers of technology for care

	Facilitators	Barriers
Informal caregiver	•‘*secure environment’* •‘*cost effective care’* •‘*greater freedom*’ •‘*improve knowledge’* •‘*privacy and independence’* •‘*a quick source of getting assistance if they fall*’ •‘*very reassuring’ especially at night* •Mobile phones: ‘*keep (family) in continual contact’*	•(tenants) ‘*may not have understanding why or how to use it e.g. wearable pendant, intercom system technology can go wrong. Needs to be closely monitored*’ •technology was ‘*not much use’* •‘*the alarm watches, not sure with dementia you remember or are able to press button depending on how severe dementia is*’
Formal caregiver	•‘*workload more manageable’* ‘*alerting staff to an incident’* •‘*it would be really hard to care without the use of technology’* •••••••‘*technology has a big impact as we can speak to tenants in rooms *etc. *without being their which assists with making sure tenants are safe’* •‘*offering more independence and security’* •‘*reassurance*’ •‘*let me know if someone needs help*’, •enhance ‘*communication*’ •provide ‘*quicker support’* •‘*give the best support for those who need it’* •‘*be aware of what is happening in places without being there*’	•‘*take away from basic effective techniques and approaches*’ •‘*lots of calls at the same time’* •‘*black areas’* where technology doesn't work in the housing scheme •‘*false alarms or volume of alarms can be distracting*’ •‘*difficult for individuals to remember how to use it*’

A total of 55% (*N* = 11) family and friend respondents felt tenants should have access to internet, 25% (*N* = 5) should not and *N* = 4 20% didn't know. It was also considered an asset to support tenant’s socialisation and families had installed internet in their relatives flat already. Interestingly, 77.42% (*N* = 24) of staff felt tenants should have access to internet, 3.23% (*N* = 1) should not and *N* = 6 (19.35%) didn't know. Responses against access to internet were around safeguarding issues for both caregivers. Attitudes towards to use of technology were captured and outlined in Table 6.

**Table 6:** Caregivers attitudes towards technology within the housing scheme

Question	Respondents	Strongly agree	Agree	Neither agree or disagree	Disagree	Strongly disagree
**Technology enables the tenant to be more independent in their own living environment**	IC (*N* = 20)	20% (*N* = 4)	40% (*N* = 8)	35% (*N* = 7)	0% (*N* = 0)	5%(*N* = 1)
	FC (*N* = 31)	48.39% (*N* = 15)	38.71% (*N* = 12)	12.9% (*N* = 4)	0% (*N* = 0)	0% (*N* = 0)
**A tenant living in the scheme has less human (face-to-face) contact with staff because of technology**	IC (*N* = 20)	0% (*N* = 0)	15% (*N* = 3)	15% (*N* = 3)	45% (*N* = 9)	25% (*N* = 5)
	FC (*N* = 31)	9.68% (*N* = 3)	9.68% (*N* = 3)	16.13% (*N* = 5)	32.26% (*N* = 10)	32.26% (*N* = 10)
**Technology enhances the quality of care provided in the scheme**	IC (*N* = 20)	15% (*N* = 3)	60% (*N* = 12)	20% (*N* = 4)	5% (*N* = 1)	0% (*N* = 0)
	FC (*N* = 31)	41.94% (*N* = 13)	45.15 (*N* = 14)	12.9% (*N* = 4)	0% (*N* = 0)	0% (*N* = 0)
**Technology reduces my relative/ friend's/ tenants privacy**	IC (*N* = 20)	0% (*N* = 0)	0% (*N* = 0)	15% (*N* = 3)	70% (*N* = 14)	15% (*N* = 3)
	FC (*N* = 31)	6.45% (*N* = 2)	9.68% (*N* = 3)	22.58% (*N* = 7)	38.71% (*N* = 12)	22.58% (*N* = 7)
**Each tenant should consent to the use of technology**	IC (*N* = 20)	15% (*N* = 3)	30% (*N* = 6)	30% (*N* = 6)	20% (*N* = 4)	5%(*N* = 1)
	FC (*N* = 31)	25.81% (*N* = 8)	51.61% (*N* = 16)	9.68% (N = 3)	6.45% (N = 2)	6.45% (*N* = 2)
**I trust the use of technology in the care of people living with dementia**	IC (*N* = 20)	15% (*N* = 3)	60% (*N* = 12)	15% (N = 3)	5%(N = 1)	5%(*N* = 1)
	FC (*N *= 31)	29.03% (*N* = 9)	41.94% (*N* = 13)	16.13% (*N* = 5)	12.9% (*N* = 4)	0% (*N* = 0)
**The tenant’s safety is more important than their right to privacy**	IC (*N* = 20)	20% (*N* = 4)	60% (*N* = 12)	10% (*N* = 2)	10% (*N* = 2)	0% (*N* = 0)
	FC (*N* = 31)	22.58% (*N* = 7)	38.71% (*N* = 12)	22.58% (*N* = 7)	9.68% (*N* = 3)	6.45% (*N* = 2)
**Safety and security of the tenant is not increased through the use of technology**	IC (*N* = 20)	5%(*N* = 1)	20% (N = 4)	10% (*N* = 2)	45% (*N* = 9)	20% (*N* = 4)
	FC (*N* = 31)	6.45% (*N* = 2)	9.68% (*N* = 3)	16.13% (N = 5)	48.39% (*N* = 15)	19.35% (*N* = 6)
**The use of technology has no benefit in supporting the tenant**	IC (*N* = 20)	0% (*N* = 0)	15% (*N* = 3)	15% (*N* = 3)	45% (*N* = 9)	25% (*N* = 5)
	FC (*N* = 31)	3.23% (*N* = 1)	6.45% (*N* = 2)	9.68% (*N* = 3)	58.06% (*N* = 18)	22.58% (*N* = 7)

*IC* Informal caregiver, *FC* Formal caregiver

## Discussion

Global health priorities want to address the care needs for the growing number of people living with dementia. The preference is for people to age in place with wrap around, person centred care that supports choice, autonomy, and independence, while complimenting the care received from formal and informal caregivers. This challenge has the potential to be supported through technology innovation and alternative care models. It is important to reflect on the entire dementia journey and explore the changing needs people have across the continuum of care. This present study examines technology used to compliment person centred care in supported living environments from three stakeholder groups; tenants, informal caregivers and formal caregivers. From a critical dementia lens, the findings demonstrated that technology enabled people that were no longer able to live in their own home, to have privacy, independence, and autonomy in a supported living setting. Previous research has indicated that supported housing can lack the resources and facilities to support the maintenance of independence and autonomy [32], technology could be the solution to support the resources required to empower tenants to live independently for longer and enable citizenship and social connectedness [11]. Finding the ethical balance between independent living and safety could be provided by digital monitoring technologies to meet the needs of all stakeholders [39].

The main findings from the technology audit highlighted a wide range of technologies and devices operational within the technology enriched settings. In line with Gibson and team’s [8] review, the technology provision across the housing schemes was fragmented and often bespoke design, meaning that no two facilities operated in the same way. It was evident that schemes operated different policies around the free movement of tenants, as some environments were locked while in others, tenants held the key to the front door. Data from the technology was not widely used within the schemes as only two facilities reported using this information for individual care planning. Careful consideration should be given to the management and use of collected data [20]. Therefore, the results would suggest that a clear plan to integrate the use of technology into caregiving for the future is required [57]. Equally, enhanced staff training and discussion on implementation could further deepen knowledge and the use of devices and data in care delivery [58]. Technology such as sensors and wearable devices were provided according to the needs of the tenant, indicating an individual and customisable approach, which is a key feature of person-centred care according to the literature [33]. Shared learning across these facilities should be promoted to inform the ongoing operational delivery of care particularly on topics like GDPR and embedding data within notes and records to evidence how pervasive technologies inform care.

Mobile phone use was commonly reported and supported feelings of connectedness within the tenant interviews. This is in line with the findings of previous research[11]. The findings highlighted very limited use of digital technology within the supported living environment overall. Opportunities for social interaction, social robots and gaming technologies can provide a more holistic approach to dementia care [34]. Digital illiteracy is a significant barrier for this population[23]. There is huge potential to harness the power of everyday technologies, particularly with a more tech savvy generation that could be living with dementia in the future. Covid-19 has changed this landscape, research has demonstrated that with support from formal carers digital technologies for connectedness can support those with low digital literacy levels [44]. The findings support the mixed economy approach to technology provision where off the self-solutions compliment state provision [59].

The interview findings indicated that tenants felt a sense of security and connectedness from the technology in the surrounding environment. These are essential features for maintaining a sense of identity and being in a lived space [25]. Challenges included the need to remember to interact with a device and awareness of the pervasive nature of the monitoring technologies. All tenants signed up to a tenancy agreement that included consent for using monitoring technologies within the living environment. However, there are ethical issues around informed consent with loss of awareness overtime [43]. Advanced care planning post diagnosis of dementia should include information and discussion on these types of accommodation so people can have explicit consent in place and triggers identified to support the transition from home to these facilities. There was evidence that a needs-based approach was taken in term of technology use, as is a key component of assistive technology and telecare provision [6]. However, no mention of an assessment to tailor the technology provision was evident. In line with previous research [15], and the ethos of person centred healthcare provision, we recommend a comprehensive assessment of need is undertaken. Additionally, it is important to consider the tailoring of technology, can this be undertaken by staff or does a person with expert knowledge need to be a part of this assessment.

The caregiver survey findings also indicated that attitudes towards the use of technology, particularly access to Wi-Fi, are still paternalistic and further percolation around this is required so people living with dementia can enjoy the benefits of technologies for social interaction, gaming, cognitive stimulation, and leisure, rather than purely healthcare. Lessons learned from the Covid-19 pandemic demonstrated the value in digital technologies for connectedness, well-being and cognitive stimulation [11]. Once again, this element of safety, reduction of risk and protection from harm was highlighted as a core element of technology use [60]. In line with the findings of Hall and team, [40], safety was considered more important than right to privacy. The survey data captured key challenges for staff that included the high volume of calls, false alarms, and its failure to work in certain parts of the building. Only 11.11% of staff felt there was a disadvantage to using technology such as tenants not remembering how to use it and that it could go wrong. These fears were very much consistent with previous research [61].

A limitation of this study was a focus on supported living in a single country in the United Kingdom. It is difficult to know if experiences are transferable across to other settings in different regions. Particularly given the unique nature of technology provision across this single country. Response bias could be present in the data as scheme managers identified the tenants to engage in the recruitment process for interviews. The low response rate for the survey could have been because of caregiver’s lack of awareness of these activities even with electronic and hardcopy distribution. The housing schemes had capacity challenges and it is possible this resulted in only half undertaking the follow up phone call to complete part B of the technology audit. The use of technology in this living space by multiple stakeholders is complex. Future research using a longitudinal ethnographic methodological approach is recommended to future explore these relationships. In addition, as the digital literacy of people moving into supported housing changes, it would be interesting to explore the capability of everyday technologies to support the caregiving relationship from diagnosis and throughout the continuum of care and changing housing models.

The present study provides new insight into stakeholder’s experiences of living, working and caregiving alongside technology in supported living environments. It was evident from the literature that choosing the correct technology to support people living with dementia in supported living environments was multifaceted. The current study highlighted the diversity of technology provision and often opportunities for housing schemes to learn from each other were overlooked. Elements of personalised care and person-centred practice were apparent with customised approaches to technology provision. Where tenants were aware they were living alongside technology, it provided a sense of security and connectedness. These findings highlighted the importance of advanced care planning and technology assessments earlier in a person’s dementia journey. Equally, providing support to harness everyday technology to sustain ageing in place and support caregiving is important. The findings also indicated that attitudes towards the use of technology, particularly access to Wi-Fi, are still paternalistic and further percolation around this is required so people living with dementia can enjoy the benefits of technologies for social interaction, cognitive stimulation, gaming, and leisure, rather than purely healthcare.

## Supplementary Information

**Additional file 1.**

**Additional file 2.**

## Data Availability

The datasets generated and analysed during the current study are not publicly available due to protecting the privacy of participants but are available from the corresponding author on reasonable request.
