# Supplementary material for: Stakeholder’s experiences of living and caring in technology-rich supported living environments for tenants living with dementia
Source: BMC Geriatr. 2023 Feb 1;23:62. doi: 10.1186/s12877-023-03751-2 (PMC9889957; doi:10.1186/s12877-023-03751-2)
Supplement: Supplementary file 1 — Additional file 1. [file 12877_2023_3751_MOESM1_ESM.docx]

**TESA-DRI: Technology Audit for Housing Schemes Part 1**

Thank you for taking the time to complete this technology audit. This is part of the research we are completing on what it is like to live and work here. Please do give expansive answers to the questions. You are welcome to attach an additional sheet and as many comments as you think would be helpful to us.

**How do you describe the technology used within your housing scheme?**

_________________________________________________________________________________________

_________________________________________________________________________________________

_________________________________________________________________________________________

_________________________________________________________________________________________

**Within the overall housing scheme**

1) How do people come in and out of the scheme?

|  | **Electronic code into keypad** | **Fingerprint enabled** | **Key card** | **No free movement** | **Other**  (Please specify) |
| --- | --- | --- | --- | --- | --- |
| **Staff** |  |  |  |  |  |
| **Tenants** |  |  |  |  |  |
| **Family** |  |  |  |  |  |

2) Are there sensors on the door(s) to alert when a person comes in/ leaves the scheme? (Please circle)

Main reception door Yes No

Side door Yes No

Doors to tenants flats/rooms Yes No

Other (Please specify) _____________________________________

Is data from these sensors gathered on a database? Yes No

3) Is there a video feed at the entrance to the housing scheme? (Please circle)

Yes No

4) Do you have any video feeds within the housing scheme? (Please circle)

Yes No

If yes please give details _________________________________________________________________

5) Information management system: Do you use: (Please circle)

Electronic notes Yes No

Hardcopy notes Yes No

A combination Yes No

Other or not sure (Please specify)_____________________________________________________

____________________________________________________________________________________________

**Alarm triggers**

6) When a tenant needs to call for help in an emergency scenario they do this through: (Please circle)

A wearable device Wall fixed buttons Pull cord Other ___________________

7) Is this alarm the same when they are in or outside their flat/ bungalow/ room?

Yes No

8) How does the member of staff receive this alert? _________________________

_________________________________________________________________________________

9) Does the tenant get an immediate response to know that a staff member received the alert? (i.e. Speech message, intercom or communication with staff) (Please circle)

Yes No

Please give details _________________________________________________________________

10) How do tenants seek non-emergency assistance?

_________________________________________________________________________________________

_________________________________________________________________________________________

**Within individual Flats or Bungalows**

11) Is there a video feed at the entrance to the tenants flat or bungalow?

Yes No

12) Is there a speaker or communication system within the tenants flat or bungalow to central hub/ nursing station/ staff pager?

Yes No

13) What technology devices are used within the scheme? (Please tick)

| **Alarms** | **Tenants flat/ home** | **All living environments** | **Customised areas** | **Common areas** | **Not within scheme** |
| --- | --- | --- | --- | --- | --- |
| Fall alarm/ detector |  |  |  |  |  |
| Smoke alarm |  |  |  |  |  |
| Flood alarm |  |  |  |  |  |
| Carbon monoxide detector |  |  |  |  |  |
| Heat/ temperature extreme alarm |  |  |  |  |  |
| Gas detectors |  |  |  |  |  |
| **Sensors** | **Tenants flat/ home** | **All living environments** | **Customised areas** | **Common areas** | **Not within scheme** |
| Motion sensors |  |  |  |  |  |
| Inactivity sensors |  |  |  |  |  |
| Automatic/ motion sensitive lights |  |  |  |  |  |
| Water temperature |  |  |  |  |  |
| Pressure sensors: Bed |  |  |  |  |  |
| Pressure sensors: Chair |  |  |  |  |  |
| Pressure sensors: Floor |  |  |  |  |  |
| Pressure sensors: Other (Please specify) |  |  |  |  |  |
| Door sensors |  |  |  |  |  |
| Enuresis/ continence sensor |  |  |  |  |  |
| **Monitors** | **Tenants flat/ home** | **All living environments** | **Customised areas** | **Common areas** | **Not within scheme** |
| Water flow control |  |  |  |  |  |
| Cooker monitor (turn off device) |  |  |  |  |  |
| Close-circuit TV |  |  |  |  |  |

14) What wearable devices are used within the scheme?

|  | All tenants | Specified tenants | No tenants |
| --- | --- | --- | --- |
| Electronic tracking device (GPS) |  |  |  |
| Alarm pendent |  |  |  |
| Alarm bracelet |  |  |  |
| Other please specify: |  |  |  |

15) What other devices are found within the housing scheme?

|  | All tenants | Specified tenants | No tenants |
| --- | --- | --- | --- |
| Easy to use telephone |  |  |  |
| Mobile phone |  |  |  |
| Music player |  |  |  |
| TV |  |  |  |
| iPad |  |  |  |
| Computer |  |  |  |
| Ease to use remote control with large buttons |  |  |  |
| Memory AID |  |  |  |
| Audio/ Audio-visual prompts |  |  |  |
| Companion robots or electronic toys |  |  |  |
| Light therapy |  |  |  |
| Electronic calendars |  |  |  |
| Other please specify: |  |  |  |

16) Would you be aware of any tenant(s) that use any other electronic assistive devices and if so can you list what these are?

_________________________________________________________________________________________

_________________________________________________________________________________________

_________________________________________________________________________________________

_________________________________________________________________________________________

19) How do staff receive alerts and information from the system?

|  | **Yes** | **No** |
| --- | --- | --- |
| **Monitoring station** |  |  |
| **Staff pager** |  |  |
| **Web application** |  |  |
| **Mobile phone** |  |  |
| **Tablet** |  |  |
| **Other (please specify)** |  | |

20) If the computer software that manages the data from the sensors and alerts has a name, can you tell us what it is?

_________________________________________________________________________________________

21) Do you keep the data from the sensors and technology?

Yes No

22) How often is data sent from sensors?

________________________________________________________________________________________

23) The data is transmitted through:

| **Network** | **Yes** | **No** | **Don’t know** |
| --- | --- | --- | --- |
| Wireless |  |  |  |
| Hardwired |  |  |  |
| 4G |  |  |  |
| Passive Infrared |  |  |  |
| Zigbee |  |  |  |
| Radio frequency transmitter |  |  |  |
| Power line interface |  |  |  |

24) Do staff access the sensor and technology data?

Yes No

If staff access the data, please indicate how this is done:

|  | **Yes** | **No** |
| --- | --- | --- |
| **Monitoring station** |  |  |
| **Staff pager** |  |  |
| **Web application** |  |  |
| **Mobile phone** |  |  |
| **Tablet** |  |  |
| **Is not accessed** |  |  |

25) How often is data accessed by staff?

________________________________________________________________________________________

26) Do you use the data from sensor and technology to reflect on the tenant and input into care plan?

Yes No

27) The format of the data on the system is illustrated in: (Please circle)

A readable format (Automatically analysed) Needs to be analysed

28) Can the data give information on:

|  | **Yes** | **No** |
| --- | --- | --- |
| Individual tenant’s physical activity level |  |  |
| Give an indication of changes in health status |  |  |
| Monitor Sleep/ wake pattern |  |  |
| **Other (Please specify)** |  |  |

If staff can set upper or lower limits (parameters) on the system for when alerts will be sent please answer these questions.

29) Can parameters change on devices to be active at certain times of the day and off at other times?

Yes No

Please give details _________________________________________________________________

30) Are members of staff able make adjustments to these parameters to change when the device is active or not?

Yes No

Please give details _________________________________________________________________

31) If you have any other comment please feel free to leave it here.

_________________________________________________________________________________________

_________________________________________________________________________________________

_________________________________________________________________________________________

_________________________________________________________________________________________

Thank you for taking the time to complete this technology audit. In the near future we would ask that you take the time to undertake a telephone interview to complete part two of this technical audit. If you have any further questions please feel free to contact XXX

*This material was developed within Ulster University and Engage with Age in the TESA-DRI project. The project was funded by the Health and Social Care Research and Development Division Public Health Agency and Atlantic Philanthropies (COM/4955/14).*
