# Supplementary material for: Stakeholder’s experiences of living and caring in technology-rich supported living environments for tenants living with dementia
Source: BMC Geriatr. 2023 Feb 1;23:62. doi: 10.1186/s12877-023-03751-2 (PMC9889957; doi:10.1186/s12877-023-03751-2)
Supplement: Supplementary file 2 — Additional file 2. [file 12877_2023_3751_MOESM2_ESM.docx]

**TESA-DRI: Technology Audit for Housing Schemes Part 2**

Thank you for taking the time to complete this technology audit. This is the second part of the technology audit that we will complete over the phone at a time and date that suits you.

1) What security procedures are in place to protect the confidentiality of the data?

________________________________________________________________________________________

________________________________________________________________________________________

________________________________________________________________________________________

2) Can you outline the security measures in place when the system is accessed?

________________________________________________________________________________________

________________________________________________________________________________________

________________________________________________________________________________________

3) Is it possible to request reports for different information needs of staff for example can the manager downloaded an overall activity profile for the scheme or could a specific resident?

________________________________________________________________________________________

________________________________________________________________________________________

________________________________________________________________________________________

4) Is the data presented in a way that makes sense to: (Please circle)

All staff trained staff managers no staff

5) Data is compiled into: (Please circle)

Reports Individual care plans Is not accessed/ used

Please give details _________________________________________________________________

6) Have staff received training on the use of technology within the scheme?

Yes No

Please give details _________________________________________________________________

7) Is the tenant asked to consent to the use of technology in their environment?

Yes No

Please give details _________________________________________________________________

8) Is the family asked to consent to the use of technology in their relatives environment?

Yes No

Please give details _________________________________________________________________

9) Please describe the process of introducing the use of technology to a new tenant.

_________________________________________________________________________________________

_________________________________________________________________________________________

_________________________________________________________________________________________

_________________________________________________________________________________________

10) Please describe the policy and governance involved in the use of technology within the housing scheme.

_________________________________________________________________________________________

_________________________________________________________________________________________

_________________________________________________________________________________________

_________________________________________________________________________________________

11) Was the technology within your scheme a bespoke system that was commissioned or is it commercially available? (Please circle)

Bespoke system Commercially available

12) If you have any other comment please feel free to leave it here.

_________________________________________________________________________________________

_________________________________________________________________________________________

_________________________________________________________________________________________

_________________________________________________________________________________________

Thank you for taking the time to complete this technology audit. If you have any further questions please feel free to contact XXX.

*This material was developed within Ulster University and Engage with Age in the TESA-DRI project. The project was funded by the Health and Social Care Research and Development Division Public Health Agency and Atlantic Philanthropies (COM/4955/14).*
